# Insights into the Microscopic Oil–Water Flow Characteristics and Displacement Mechanisms during Waterflooding in Sandstone Reservoir Rock Based on Micro-CT Technology: A Pore-Scale Numerical Simulation Study

**DOI:** 10.3390/ma16093555

**Published:** 2023-05-06

**Authors:** Bingtao Hu, Guoliang Chai, Xingjun Liu, Xiaofeng Wen, Zhaolin Gu, Liaobo Xie, Shifeng Han, Junwei Su

**Affiliations:** 1 School of Human Settlements and Civil Engineering, Xi’an Jiaotong University, Xi’an 710049, China; hubingtao@stu.xjtu.edu.cn (B.H.);; 2 Changqing Division, China Petroleum Logging Co., Ltd., Xi’an 710201, China

**Keywords:** porous materials, waterflooding, porous media flow, microscopic numerical simulation, micro-CT technology

## Abstract

The low oil recovery rate observed in current oil fields is largely attributed to the presence of remaining oil trapped in the pores of porous media during waterflooding. To improve the recovery rate, it is imperative to gain an understanding of the oil–water flow characteristics and displacement mechanisms during waterflooding, as well as to elucidate the underlying mobilization mechanisms of residual oil at the pore scale. In this paper, we explore these issues in depth by numerically investigating the influence of factors such as water injection velocities, oil–water viscosity ratios, and wettability conditions on pore-scale oil–water flow characteristics and oil recovery rate. To this end, we employ a direct numerical simulation (DNS) method in conjunction with the volume of fluid (VOF) method to study the microscopic displacement mechanisms of waterflooding in a reconstructed two-dimensional digital rock core based on micro-CT technology. In addition, the particle tracing method is adopted to identify the flow path and dominant areas during waterflooding in order to mobilize the residual oil within the pores. The findings indicate that the oil–water flow characteristics in porous media are determined by the interplay between capillary and viscous forces. Furthermore, the oil recovery rate is 10.6% and 24.7% lower under strong water-wet and oil-wet conditions than that (32.36%) under intermediate wettability conditions, and the final oil recovery rate is higher under water-wet conditions than under oil-wet conditions. The seepage path and the dominant areas are directly linked to the capillarity formed during waterflooding. The findings of this study are significant in terms of enhancing the recovery rate of residual oil and provide a novel perspective for understanding the waterflooding process.

## 1. Introduction

Conventional fossil fuels, namely oil, coal, and natural gas, currently account for 82.3% of global energy consumption, with oil alone representing 30.9% [1]. Although vital to human civilization and economic development, crude oil reserves are limited, with proven reserves of 1.73 trillion barrels as of the end of 2019 and a lifespan of approximately 49.9 years at current production rates [1]. Waterflooding technology is widely employed in oil production as a secondary recovery method, but it will ultimately lead to insufficient crude oil production rates and pose significant challenges for further oil extraction, particularly in low-permeability, low-pressure, low-temperature, and low-yield reservoirs [2]. Approximately 60% of the original crude oil remains unrecovered during the final stage of waterflooding exploitation, and the residual oil within the rock presents in various forms (irregular strings, streaks, squirts, and discontinuous oleic saturations) [3,4,5]. Although various enhanced oil recovery technologies, including chemical flooding, thermal flooding, and combination flooding, are continuously emerging to boost the oil recovery efficiency [3,4,5], elucidating the fundamental mechanisms underlying residual oil formation is paramount before adopting tertiary oil recovery methods.

Residual oil formation in reservoirs can be attributed to two primary reasons. The first reason is the inherent reservoir heterogeneity and the application of various displacement technologies, resulting in the entrapment of crude oil within the unswept rock regions [6,7,8]. The second reason is the abrupt variations in pore structure, rock wettability, and fluid stress status, which can also contribute to residual oil that remains within the swept area [7,9,10,11,12,13,14,15]. A series of studies revealed that the pore structure and wettability of porous media play a pivotal role in controlling two-phase immiscible displacement patterns, residual oil formation, and the rate of oil recovery rate [7,8,9,10]. Further, the capillary barrier effect, resulting from the abrupt change in geometry between the minor and major empty regions of porous media, is the fundamental cause of residual oil formation [16,17,18,19,20]. Here, the capillary barrier effect is defined as a phenomenon where the invading fluid stops advancing until the waterflooding pressure exceeds a specific value to induce fluid motion. However, current investigations [16,17,18] concerning the capillary barrier phenomenon are phenomenological, and an accurate and exhaustive characterization of the pore-level flow dynamics quantitatively within porous media is essential for mobilizing the trapped oil in pore spaces.

Oil reservoir exploitation is a complex process that involves multiscale, multiphysics, and multiphase flows that are significantly influenced by various factors, such as pore structure characteristics, fluid properties, flow conditions, among others [21,22,23,24,25,26,27,28,29]. The intricate pore structure of the reservoir cores, which comprises irregular pore space and narrow throat channels connecting different pore spaces, leads to several specific pore-scale events in porous media flow, including drainage [4,7,9,14,30], imbibition [17], pore body filling [14,16,17,31], snap-off [32,33], Haines jump [34], and capillary barrier [11,16,17,18,19,20]. Physical visualization experiments and pore-scale numerical simulation are two mainstream approaches [11,35,36,37,38,39,40,41,42,43,44,45,46,47] to describe two-phase flow dynamics in porous media at Darcy scale and pore-level scale. Physical experiments based on miniature core flooding platform, microfluidic chip technology, and micro-CT imaging technology [9,12,30,48,49,50,51,52,53,54] can reproduce the macroscopic immiscible flow in the rock core via visualizing the microscopic flow process in pore space and describing the microscopic immiscible flow characteristics from a phenomenological view, which is extensively used to study the impact of various factors, including fluid physicochemical property, rock inhomogeneity, wettability, displacement modes, and pore structure on oil recovery rate and displacement mechanisms. Zhao [12] and Sharma [30] investigated the influence of wettability and geometry heterogeneity on immiscible fluid flow using microfluidic imaging technology and Micro-PIV technology. Furthermore, Gao [49,50] performed a steady-state laboratory-scale core flooding test on carbonate rock with mixed-wet regimes and determined the capillary pressure by analyzing the microscopic images captured via X-ray microtomography scanning technology. However, due to the resolution limitations of optical microscopy and the difficulty of replicating natural pore structures of rock, physical experiments are unable to reveal the underlying displacement mechanisms of waterflooding. Darcy’s law characterizes the linear correlation between pressure gradient and flow rate in porous media at the macroscopic scale and is valid for Reynolds numbers between 1 and 10 [55,56]. However, when the flow rate increases, the law scales non-linearly and may transition to a power law if the displacement pressure is insufficient to overcome the capillary barrier effect [57,58]. It is challenging to directly correlate microscopic properties with averaged parameters at Darcy scale [59]. Therefore, high-resolution imaging technologies and advanced computational methods play a crucial role in bridging the gap between the microscopic pore-level scale and macroscopic scale. Numerical simulations based on Darcy’s law at the macroscopic scale fail to accurately capture the intricate details of the microscopic oil–water process. Consequently, pore-scale numerical simulation approaches, classified into direct numerical simulation (DNS) and pore network modeling (PNM), have become increasingly prevalent in characterizing complex flow processes in porous media at pore scale. The emergence of X-ray microtomography technology allow us to visualize and characterize the oil–water saturation distribution and capture pore-scale events in two-dimensional μ-CT slice images under different wettability regimes, which enables us to gain a profound comprehension of the pore-scale waterflooding mechanisms. Raeini [35] focused on numerical investigation of layer flow and snap-off, while Wang [36] discovered that intermediate-wet or weak water-wet rock characteristics positively affect oil recovery in rocks when considering wettability. Similarly, Yang [11] numerically analyzed the residual oil in a reconstructed porous model using micro-CT technology, accounting for the effects of capillarity and wettability. Additionally, Saraf [38] reviewed state-of-the-art advances in pore-scale numerical simulation and CT scanning techniques that aim to describe sequestered CO_2_. Nowadays, a pore-scale network model and a three-dimensional digital rock core model can be reconstructed using high-resolution X-ray microcomputed tomography technology, which facilitates the three-dimensional simulation of oil–water flow in porous media [39]. Capturing pore-scale events of porous flow within a reconstructed digital rock using direct numerical simulation (DNS) is both computationally expensive and time-consuming, especially for three-dimensional porous media flow [60,61]. While pore network modeling (PNM) is computationally efficient and applicable to large-scale porous media, its idealized pore space geometry may not accurately represent complex flow processes [62]. A finite-volume-method-based DNS approach is adopted in this paper to characterize more detailed flow characteristics and physical quantities of pore-scale events of the capillary barrier phenomenon. Two-dimensional pore simulation of porous media flow combined with micro-fluidic platform is still the mainstream approach to investigate the microscopic mechanisms of waterflooding, and the two-dimensional physical rock model extracted from a single slice of 3D digital rock can preserve most of the characteristics of the 3D natural rock, such as particle shape, pore-throat network, and connectivity, which has been widely applied to study the two/three-phase flow in natural and technological processes, such as enhanced oil recovery, geological carbon sequestration, and remediation of non-aqueous phase liquids. Despite the existence of extensive investigations on porous media flow and the waterflooding process in recent years, with consideration afforded to capillarity and wettability, the existing studies are limited to qualitative analysis and phenomenological characterization of the dynamic behavior. The underlying microscopic mechanisms of waterflooding, particularly the remaining oil mobilization mechanisms due to the capillary barrier effect, remain poorly understood and have not been fully elucidated in the available literature.

The primary objective of this study is to address the current limitations in the existing research by elucidating the microscopic mechanisms governing the flow behavior and mobilization of trapped oil in the pores during waterflooding to some extent based on our prior research [18]. In this paper, special attention is devoted to the mechanical characteristics of capillary barriers, and, based on this, influences of capillary pressure barriers on flow behaviors under different injection conditions, viscosity ratios, wetting conditions, seepage paths, and dominant areas are systematically studied so as to elucidate the pore-scale flow dynamic process from a mechanical perspective.

## 2. Materials and Mathematical Model

### 2.1. Sandstone Sample

**Figure 1:**
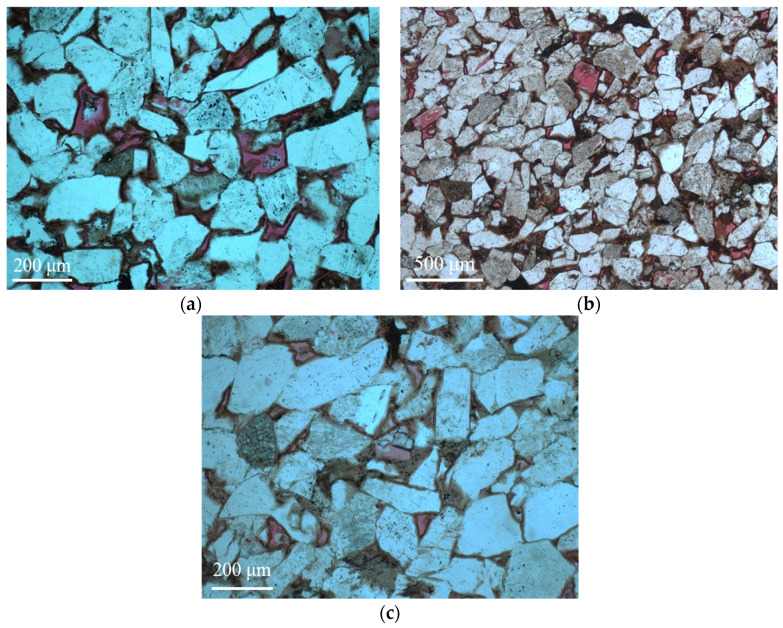
The casting thin section of the sandstone reservoir rock core: (**a**) developed chlorite film and intergranular pores; (**b**) developed intergranular pores, solution pores, and feldspar fractures; (**c**) authigenic garnets in developed solution pores.

The natural reservoir core sample was selected from Chang 6 member with depth of 1421.82 m in Yanchang oil field, Shaanxi Province, China. After oil washing, salt washing, and drying, the casting thin section was prepared by injecting vacuum-impregnated red epoxy resin into core sample and its images are captured by the optical microscope. Figure 1 shows the casting thin section images of the rock core. The mineral composition, mineral content, grain size, and pore structure are identified from the characteristics of the casting thin sections. Here, the reservoir rock core is identified as a type of fine feldspathic sandstone. The identification results of the casting thin section revealed that the lamellar minerals in the sample are well aligned. The thickness of the film-like chlorite is 3–5 μm and the chlorite is uniformly distributed. Calcite cement fills the pores and autogenetic quartz similar to horse teeth grows in the perpendicular direction of the grain surface. Poor connectivity of the pores and rare fractures formed by the compaction behavior of the feldspar are observed. The formed fractures can play a role in connecting the pores, which is often observed in pores of the feldspar cements. When compared to sandstone rock, carbonate rocks exhibit a more intricate geometry due to the presence of vugs and variations in porosity and permeability between layers [50]. Additionally, these rocks tend to be oil-wet, which further complicates their properties. The highly heterogeneous nature of carbonate rocks, combined with their complex pore structure, can result in ineffective hydrocarbon production [3].

### 2.2. Digital Rock Reconstruction

**Figure 2:**
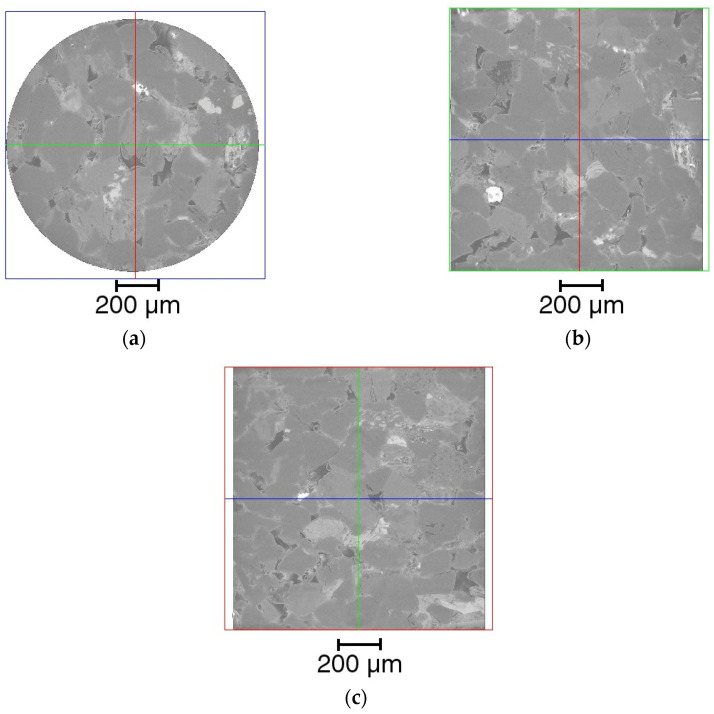
Grayscale μ-CT image of the cylindrical core sample from the sandstone: (**a**) top view profile (X–Y plane represented by blue line); (**b**) front view profile (X–Z plane represented by green line); (**c**) side view profile (Y–Z plane represented by red line).

**Figure 3:**
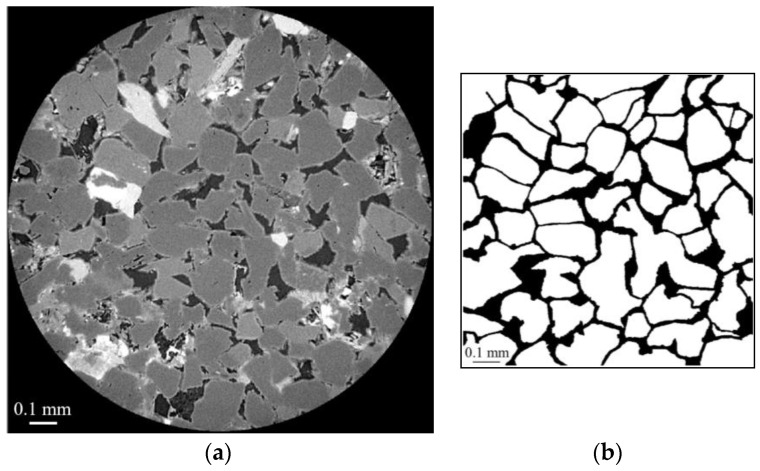
The μ-CT image of the sandstone slice sample and the 2D pore network extracted from the natural sandstone: (**a**) the gray-scaled μ-CT slice of the sandstone sample, with an image size of 2 mm × 2 mm; (**b**) the 2D pore network, with an image size of 1.44 mm × 1.44 mm.

**Figure 4:**
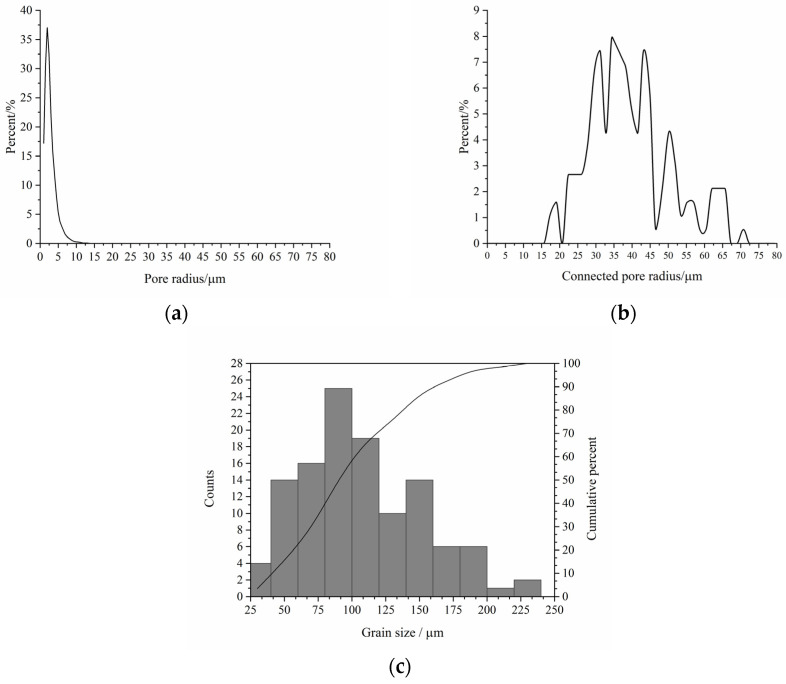
Structural properties of the sandstone sample: (**a**) pore radius distribution; (**b**) connected pore radius distribution; (**c**) cumulative distribution curve of the grain size.

X-ray micro-CT technology is commonly used to qualitatively analyze internal structures of porous materials, especially the geomaterials, and enjoys wide applications in earth science and petroleum engineering [49,50]. The main advantage of the technology is its nondestructive nature in performing the three-dimensional imaging. In this study, a cylinder with diameter of 2 mm was cut from the selected area of the cylindrical sample and scanned via the X-ray microcomputed tomography scanner (ZEISS Xradia 510 Versa, Carl Zeiss AG, Oberkochen, Germany) with spatial resolution of 0.7 μm. Figure 2 shows the grayscale μ-CT image of the cylindrical core sample from top view, front view, and side view. Figure 3 shows the μ-CT image of the sandstone slice sample and the 2D pore network extracted from the natural sandstone. To obtain the 2D pore network model, we followed a specific procedure. Firstly, the image of the thin sections was converted to a grayscale image, as shown in Figure 3a, using Image J software. Secondly, the gray-scaled thin section image was segmented into pores and grains, as depicted in Figure 3b, using Avizo software. This segmentation process led to the creation of a pore network, which was subsequently used for microscopic flow simulations of oil and water in this study. The pore radius, the connected pore radius distribution, and the cumulative distribution curve of the two-dimensional digital rock core are obtained by quantitatively analyzing the binarization images using a Python toolkit PoreSpy and ImageJ software, which are demonstrated in Figure 4. The 2D pore network model obtained from micro-CT images of rock thin sections can capture much of the geometry information of the actual rocks. Further, the size scale of the pores is comparable to that of the sandstone reservoirs, making it a well-suited and ideal representation of the in situ structure for the numerical simulation and physical observation experiments of the two-phase immiscible displacement.

### 2.3. Mathematical Model for Pore-Scale Simulation of Immiscible Flow Dynamics

The macroscopic flow dynamics in sandstone reservoir rock during waterflooding are predominantly mirrored in pore-scale flow characteristics within numerous pores. Therefore, a systematic investigation of the flow dynamics and the flow characteristics within the pores is crucial for elucidating displacement mechanisms during waterflooding at microscopic scale. Numerical simulation technology offers a robust and efficient means to investigate in depth the flow process on a microscopic level. Thus, the multiphase flow characteristics, changes in flow pattern, relationships between the parameters of injection and extraction, as well as the macroscopic displacement mechanisms can be studied in terms of the spatial oil–water distribution, velocity distribution, and pressure distribution results. To this end, the mathematical model for immiscible two-phase flow is established in this paper. Here, the Navier–Stokes equation is employed to characterize the flow, and the volume of fluid method [63] is used to capture the interfaces of immiscible phases implicitly as well as to obtain spatial oil–water distribution during waterflooding. To change the contact angle magnitude measured at equilibrium, the conditions of water-wet, intermediate-wet, and oil-wet are taken into consideration [15]. Readers are referred to studies in the literature [16,21,26,27] for more details. In this section, the fundamental governing equations and case setup information are presented below.

#### 2.3.1. Mathematical Model

For incompressible flow in rigid porous media, the conservation of mass equation reads

(1) ∇·u=0

where **u** denotes the mean velocity field of the oil and water phases, m·s^−1^.

The momentum conservation equation for characterizing the oil–water flow is provided by

(2) $$\frac{\partial \rho u}{\partial t}+\nabla \cdot \left(\rho uu\right)-\nabla \cdot \left(\mu \tau \right)=-\nabla p+\rho g+F_{\sigma }$$

where ρ is average density of the two phases, kg·m^−3^; μ is average dynamic viscosity, Pa·s; p is dynamic pressure, Pa; **g** is gravity acceleration, m·s^−2^; **F**_σ_ is the interfacial tension force, kg·m^−2^·s^−2^; τ is the strain tensor rate, s^−1^.

Equation (2) indicates that the immiscible two-phase flow dynamics are governed by not only the driving force (pressure gradient) but also the viscosity μ and interfacial tension **F**_σ_. Therefore, to optimize the reservoir development, measures to modify viscosity and interfacial tension are imperative in addition to adjusting displacement pressure.

The interfacial tension force reads

(3) $$F_{\sigma }=\sigma \delta_{s}kn$$

where σ denotes the surface tension coefficient, N·m^−1^; $\delta_{s}$ denotes interface area per unit volume, m^−1^; k denotes the interface curvature, m^−1^; **n** denotes the unit vector normal to the interface.

The Laplace formula can be used to determine the capillary force (capillary pressure) for a pore with radius r, and the force is given by

(4) $$F_{\sigma }=\frac{\sigma cos\left(\theta \right)}{r}$$

where θ denotes the equilibrium contact angle, which refers to the angle formed between a solid surface and an immiscible fluid at thermodynamic equilibrium.

In low-salinity waterflooding, the contact angle is the critical factor affecting oil recovery rate, whereas the impact of salinity on interfacial tension is negligible. Thus, adjusting the contact angle becomes essential to effectively regulate and optimize the oil recovery rate.

The saturation equation for water phase reads

(5) $$\frac{\partial \alpha }{\partial t}+\nabla \cdot \left(\alpha u\right)=0$$

where α denotes the water saturation.

The objective of this study is to investigate the microscopic flow behavior of oil and water, as well as the displacement mechanisms that occur during waterflooding in sandstone reservoir rock. Meanwhile, to gain a better understanding of waterflooding in carbonate reservoirs, it is crucial to consider various factors, such as the dissolution and re-precipitation of carbonate rock minerals, chemical reactions, and salinity content. The approach proposed in this paper provides a basic framework for simulating two-phase flow in pore structures and is highly adaptable to integrate chemical reactions into the equations.

The implementation of the previously described pore-scale simulation model has been achieved in the open-source CFD software package OpenFOAM, using its inherent free interface solver interFoam for free surface simulations. The only difference between the modified solver and interFoam solver lies in the techniques employed to address the interfacial tension. The interface treatment approach proposed in our previous research [64] is adopted in this paper to eliminate the unphysical oscillation of the velocity of the conventional VOF algorithm. Equations (1), (2) and (5) are discretized using the finite volume numerical scheme to obtain a system of linear algebraic equations. The PISO algorithm is utilized to effectively separate the interdependent velocity and pressure fields, allowing for a more efficient and accurate solution of the governing equations. To validate the modified solver, its results against those obtained using the interFoam solver and the analytical solution for a standard capillary rising case are compared, and the results of the modified solver better approximated the exact solution than those of the interFoam solver. This validates the accuracy and reliability of the model for simulating pore-scale multiphase flow behavior. Interested readers can refer to our previous research papers [18,24,64] for more detailed information.

#### 2.3.2. Case Setup

**Figure 5:**
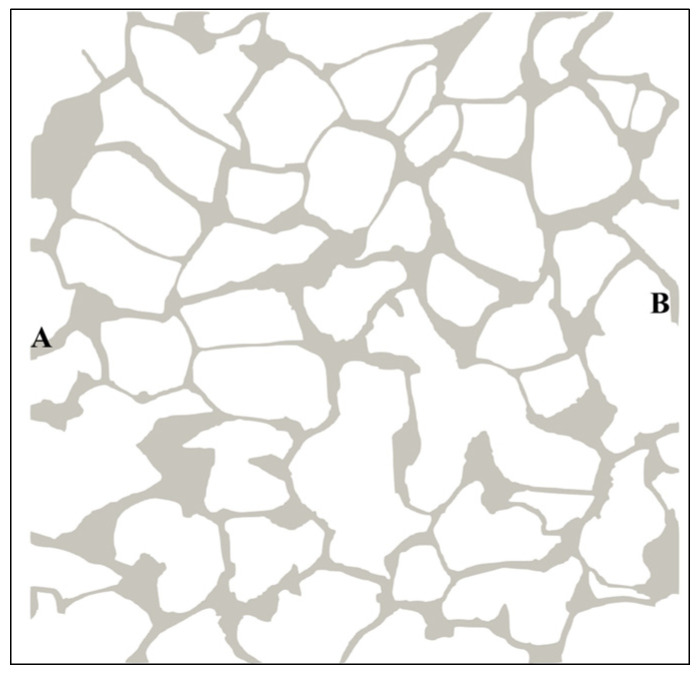
Two-dimensional digital rock core model. (A indicates the inlet and B indicates the outlet; gray color represents the brine; image size = 1.44 mm × 1.44 mm.)

The two-dimensional digital rock core model used in the paper is provided in Figure 5. The sandstone particle is represented by the white region, while the pore channels are denoted by the gray region, as shown in the figure. The digital rock core has physical dimensions of 1.44 mm × 1.44 mm and is initially saturated with oil. As to the physical model mesh, a vectorization process was first performed on the reconstructed 2D digital core physical model (Figure 5) to obtain its boundaries, and then the model was imported into the Pointwise meshing software for unstructured meshing; the mesh is composed of approximately 132,500 cells. The velocity field at the inlet, the pressure field at the outlet, and the volume fraction of the aqueous phase at the inlet were set as Dirichlet boundary conditions, while the velocity field at the outlet, the pressure field at the inlet, and the volume fraction of the aqueous phase at the outlet were set as Neumann boundary conditions. The wall of the porous medium was set as a non-slip boundary condition. The water–oil–solid three-phase contact line boundary condition was set by specifying a constant contact angle value. During the calculation process, the Gamma interpolation scheme was used to discretize the convection term of the governing equations, and the Crank–Nicolson numerical scheme was used to discretize the time term. The residual of each physical quantity was set to 10^−6^ and the Courant number was set to 0.1. The time step was initially set to 10^−6^ and was later adjusted adaptively. To conduct the simulations, we used OpenFOAM version 2.3.0 on a single AMD Epyc 7742 workstation running Ubuntu 20.04 LTS. The workstation is equipped with a 64-core processor with a dominant frequency of 2.25 GHz CPU and 128 GB of RAM. To minimize the simulation time, we utilized domain decomposition and MPI (Message Passing Interface) to perform parallel computation. A single simulation run for a given case with the aforementioned computational setup takes approximately two weeks. During the simulation, water phase is introduced into the model at port A to displace the oil out through port B. Other positions in the physical except port A and B are closed. Table 1 displays the boundary conditions, while Table 2 presents the fluid properties.

### 2.4. Methods of Obtaining the Seepage Path and the Dominant Areas during Waterflooding

The identification of seepage path and dominant fluid areas within porous media is essential for elucidating the dynamic mechanisms of waterflooding [65]. The flow path is obtained using particle tracing method, while, for the determination of the dominant areas of waterflooding, partition method of the pore space combined with the particle tracing method are used. Based on the pore space partition method proposed in this section, the core structure can be classified into several distinct single pore–throat structures. The flow characteristics of local regions of the core can be investigated by defining local statistical parameters for each partition zone and combining single pore structures into local geometries with arbitrary shape using different methods. Analysis of the simulation results employing the partition approach can aid in investigating the dependence relationship between local fluid flow characteristics and local geometric structures. Additionally, this analysis can facilitate the identification of factors that influence refined pore structures and displacement conditions on the evolution of the remaining oil distribution. For more details, readers are referred to relevant studies in the literature [21,22,23,24,34].

#### 2.4.1. Extraction Method of the Seepage Path during Waterflooding

The flow path characterized by pore network lines during waterflooding can be obtained using particle tracing method, and the procedures are as follows:

**Step 1:** The pore network of a rock core can first be constructed via extraction of its central axis using the maximum sphere method. Then, define the network grid points as the pore space, the network edges as the channels, and the positions with minimum pore radius as the throat positions. Finally, divide the pore space from the throat position based on the central axis of the pore space to form multiple pore partitions, with the throat channels being the interfaces between different partitions.

**Step 2:** For a specific waterflooding simulation case, obtain the velocity field data at the time when the seepage path needs to be extracted.

**Step 3:** Include the tracer particles with no collision volume. The particles move with the fluid velocity field, and its velocity is obtained by interpolation of the velocity field. Index sequences of all pore partitions where each tracer particle passes through are recorded in the process of the particle movement. It is worth noting that the collision of the tracer particles with the pore wall is complete elastic collision.

**Step 4:** Release the particles randomly from the inlet of the porous medium physical model and track the motion of the particles within the pore space using the Lagrangian particle tracking method until no particles flow out of the computational domain of the porous medium from the inlet.

**Step 5:** Count the index sequences of pore partitions where the particles of the computational domain flow through. Different particles may correspond to the index sequence within the same pore partition and a seepage path is shared by these different particles with the same index sequence.

**Step 6:** For a specific pore partition index sequence, a complete seepage path is finally formed by successively adding the pore network lines between the two adjacent pore partitions to the current seepage path.

#### 2.4.2. Method of Obtaining the Dominant Areas during Waterflooding

The method of identifying microscopic mobilization pores is adopted to determine the dominant areas during waterflooding, and the method involves the following steps:

**Step 1:** The step referred to is the equivalent of Step 1 outlined in Section 2.4.1.

**Step 2:** The step referred to is the equivalent of Step 2 outlined in Section 2.4.1.

**Step 3:** The step referred to is the equivalent of Step 3 outlined in Section 2.4.1.

**Step 4:** Release particles randomly from the inlet of the porous medium and the number of the particles is M. Track the motion of the particles within the pore space using the Lagrangian particle tracking method until no particles flow out of the computational domain of the porous medium from the inlet and count the number N of all particles flowing out of the computational domain as well as the number O of particles flowing through each pore partition to the outlet of the computational domain.

**Step 5:** Calculate the value of O/N and visually characterize the values of the relevant pore partitions using different colors throughout the entire computational domain.

## 3. Results and Discussion

### 3.1. Effect of Different Water Injection Velocities on the Flow Characteristics

The distribution characteristics in the two-phase system during waterflooding are investigated by injecting water into the core from port A under varying injection velocities. Velocity values of 0.001, 0.005, 0.01, 0.015, and 0.02 m/s are employed, corresponding to capillary numbers of 1.42 × 10^−5^, 7.14 × 10^−5^, 1.42 × 10^−4^, 2.13 × 10^−4^, and 2.84 × 10^−4^, respectively. The oil–water viscosity ratio is 5:1 and the contact angle measures 45°. The boundary conditions and physical properties of the fluids employed for the simulation are presented in Table 1 and Table 2.

**Figure 6:**
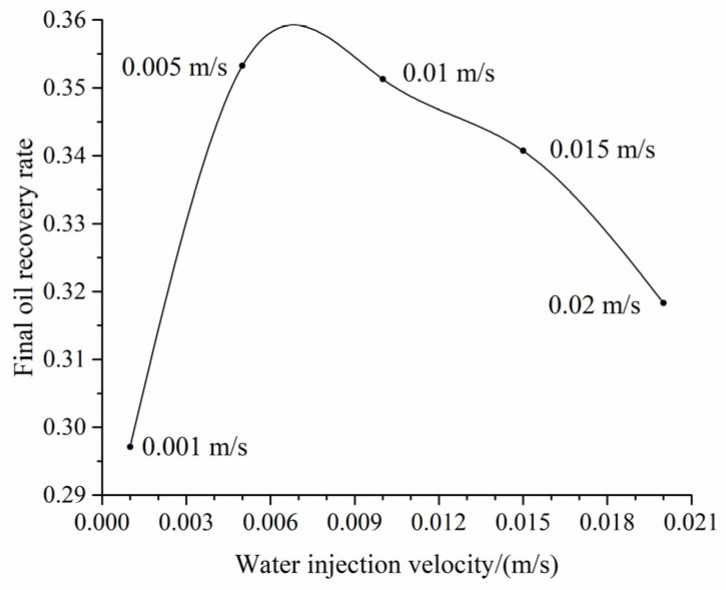
The relationship between water injection rates and the final oil recovery rate.

The relationship between water injection velocities and the final oil recovery rate is displayed in Figure 6. The figure illustrates a notable trend where the final oil recovery rate remains relatively low at lower water injection rates. As the water injection rates increase, the final oil recovery rate increases until reaching a tipping point, after which point it begins to decline. There exists an optimal water injection rate for the rock core shown in Figure 5, primarily due to the dominance of capillary effects during low-rate waterflooding. The occurrence of capillary fingering will lower the overall oil recovery rate. At higher injection rates, the viscous effect dominates and results in the decrease in the oil recovery rate due to the occurrence of viscous fingering. At intermediate injection rates, the viscous effect and capillary effect reach a state of equilibrium, facilitating a smooth advancement of the interface and ultimately resulting in the highest oil recovery rate.

**Figure 7:**
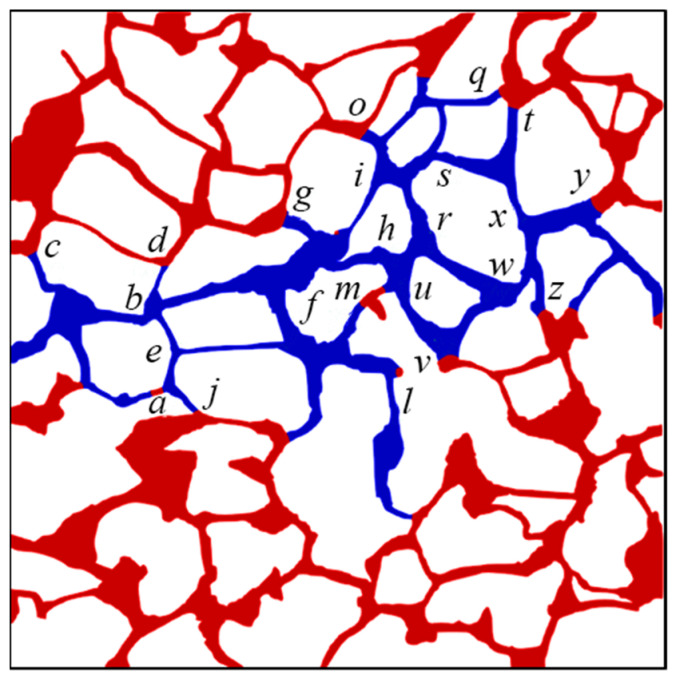
The final oil–water distribution and the corresponding interface positions. (The interface positions are designated by lowcase letters in italics; the oil and water phases are distinguished by red and blue color, respectively.)

The oil–water distribution at different moments and corresponding interface positions at certain moments are provided in Appendix A. The final oil–water distribution and the corresponding interface positions are illustrated in Figure 7, from which we can conclude that the interfaces of oil and water phase remain positioned at the intersection between the throat and pore space, where the abrupt change in pore radius occurs, as clearly indicated in the figure. Even under water-wet conditions, the capillary force maintains its resistance characteristics.

### 3.2. Effect of Different Viscosity Ratios on the Flow Characteristics

The microscopic flow characteristics under various oil–water viscosity ratios are investigated by changing the oil viscosity, and the water phase is injected into the core from port A. The viscosity ratios of 2, 5, 10, 15, and 20 are considered here. The water injection rate is established at 0.005 m/s and the contact angle is fixed at 45°. Boundary conditions and fluid properties for simulation are presented in Table 1 and Table 2, respectively.

**Figure 8:**
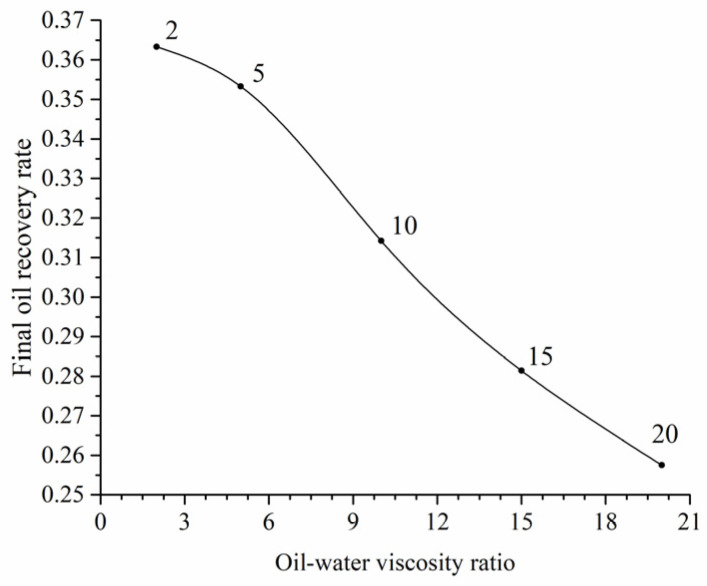
The relationship between oil–water viscosity ratio and final oil recovery rate.

The relationship between the final oil recovery rate and viscosity ratios is shown in Figure 8. A negative correlation exists between final oil recovery rate and viscosity ratios. Viscosity ratios below 5 have a minimal impact on oil recovery rate, while ratios above 5 result in a steep decline.

**Figure 9:**
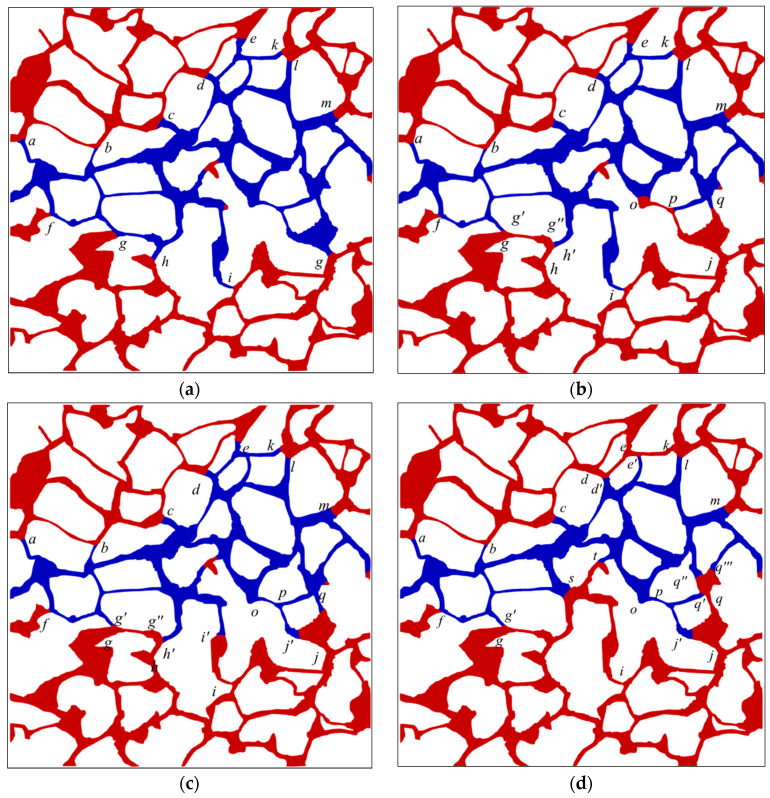
The oil and water distribution after waterflooding (Ca = 7.14 × 10^−5^) under varying viscosity ratios: (**a**) 2; (**b**) 10; (**c**) 15; (**d**) 20. (The interface positions are designated by lowcase letters in italics; the oil and water phases are distinguished by red and blue color, respectively).

The oil–water distribution after waterflooding (Ca = 7.14 × 10^−5^) under varying viscosity ratios is depicted in Appendix B for various oil–water viscosity ratios, and the phase distributions at oil–water viscosity ratios of 2, 10, 15, and 20 are displayed in Figure 9. Typically, the oil–water interfaces consistently remain located at the intersection of the pore space and throat channel on both sides of the primary water-bearing channel after waterflooding due to the occurrence of the capillary barrier phenomenon at that position, which hinders further advancement of the interface. Figure 9a illustrates the capillary barrier phenomenon at interface *a*, *b*, *c*, *d*, *e*, which results in the trapping of oil at the top left corner in the pore space. In general, the main water-bearing channel tends to become increasingly narrow as the viscosity ratio increases. For instance, in the case of an oil–water viscosity ratio of 20, residual oil remains trapped within channel *d’e’* and *e’k*, while both channels are effectively swept at other viscosity ratios. The amount of remaining oil trapped in a specific channel may vary at different viscosity ratios. The interface comes to a standstill at position *s* for channels between positions *i* and *s*, and Figure 9d provides an example of where the oil–water interface stops advancing at position s, leading to the residual oil becoming trapped. Meanwhile, the interface ultimately stops at position *i’* when the viscosity ratio is 15, with only a small amount of oil trapped in this channel, as illustrated in Figure 9c. Conversely, a low viscosity ratio is unlikely to facilitate the sequestration of residual oil in the channel.

**Figure 10:**
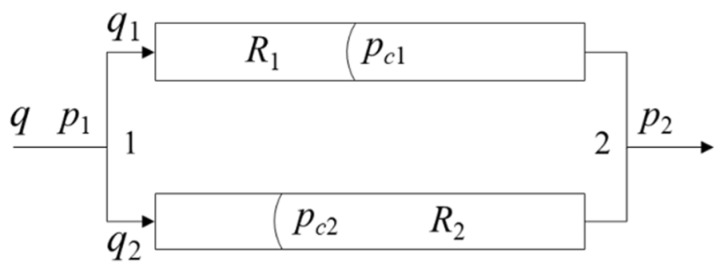
Diagram of dual-channel configuration.

The observed phenomenon is attributed to dynamic inhomogeneity ensuing from the alteration in phase distribution during waterflooding. This phenomenon can be elucidated by the parallel channels illustrated in Figure 10, where *q* denotes the total volumetric flow through parallel channels, *q*_1_ and *q*_2_ represent fractional volumetric flow allocated to channel 1 and channel 2, and *R*_1_ and *R*_2_ denote the viscous resistance of channel 1 and channel 2, respectively. Furthermore, *p*_1_ and *p*_2_ denote the pressure at point 1 and point 2, respectively, without considering the capillary effect occurring in the two parallel channels, that is, *P*_c1_ = 0 and *P*_c2_ = 0. Moreover, we assume that the viscous resistance of channel 1 *R*_1_ is less than that of channel 2 *R*_2_. Then, it can be inferred from Equations (6) and (7) that the fluid rate in channel 1 is larger than that of channel 2. As the water phase advances in both channels, the oil phase is gradually replaced, and the average viscosity of the two channels decreases. Channel 1 experiences a greater decrease in resistance due to its higher flow rate, leading to a more rapid increase in its flow rate and the viscous fingering phenomenon. The viscous fingering effect is more pronounced with higher oil–water viscosity ratios but with a narrower spatial sweep area. The sweep order of channels and oil recovery rate depend on the pressure magnitude during the displacement. As shown in Figure 9d, oil displacement occurs in channel *j’o* for viscosity ratios of 20, 15, and 2, while oil is trapped in that channel in the case of oil–water viscosity ratio 10 due to the disturbance in channel op induced by channel *i’i*, as demonstrated in Figure 9b,c. Considering the complexity of natural rock structure, the disturbance effect can be theoretically analyzed using the two parallel connected tubes.

(6) $$q_{1}=\frac{R_{2}}{R_{1}+R_{2}}q-\frac{p_{c2}}{R_{1}+R_{2}}+\frac{p_{c1}}{R_{1}+R_{2}}$$

(7) $$q_{2}=\frac{R_{1}}{R_{1}+R_{2}}q-\frac{p_{c1}}{R_{1}+R_{2}}+\frac{p_{c2}}{R_{1}+R_{2}}$$

Quantitative analysis can be conducted to study the capillary force disturbance on the parallel channels illustrated in Figure 10. Equations (6) and (7) reveal that the oil–water interface in one capillary tube significantly affects the fluid flow in its parallel channel. If an interface forms in channel 1, it increases flow velocity in channel 1 but decreases it in channel 2 due to capillary action. The presence of an oil–water interface in channel 2 will affect its parallel channel in a similar manner. Capillary force, acting as a driving force in one channel, will hinder fluid flow in its parallel channel, while capillary force with resistance characteristics in one channel will promote fluid flow in its parallel channel. The magnitude of the inhibition and promotion effects is determined by the ratio of the capillary force to the viscous resistance, which is referred to as the disturbance magnitude between the channels.

### 3.3. Effect of Different Wettability Conditions on the Flow Characteristics

The oil–water flow characteristics can be obtained by continuously injecting water into the porous media under varying wettability conditions. The contact angles adopted here are 30°, 60°, 90°, 120°, and 150°. The water injection velocity is maintained at 0.005 m/s and an oil–water viscosity ratio is fixed at 5. Table 1 and Table 2 show the boundary conditions and physical properties of the fluid.

**Figure 11:**
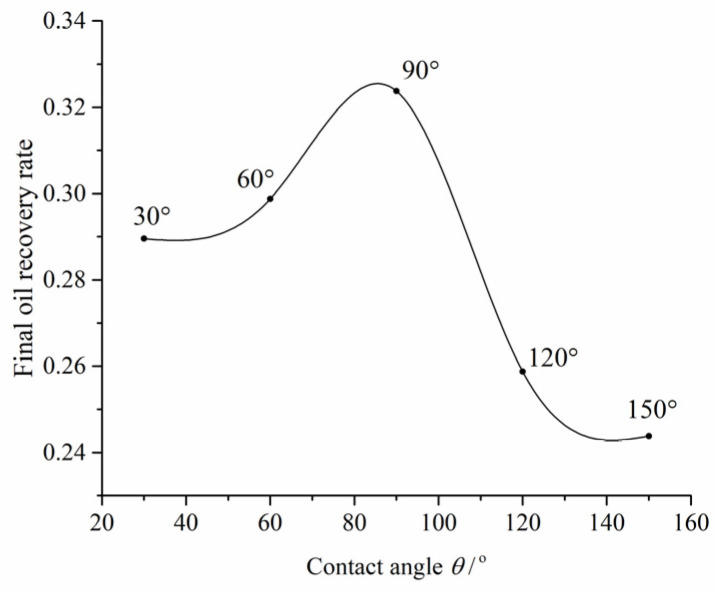
The relationship between the final oil recovery rate and contact angles.

The relationship between the final oil recovery rate and contact angles is displayed in Figure 11. As indicated in the figure, the ultimate recovery of oil is relatively lower under extreme water-wet and oil-wet conditions compared to the intermediate wettability regime. Moreover, the final oil recovery rate in water-wet conditions is relatively higher than that in oil-wet conditions.

**Figure 12:**
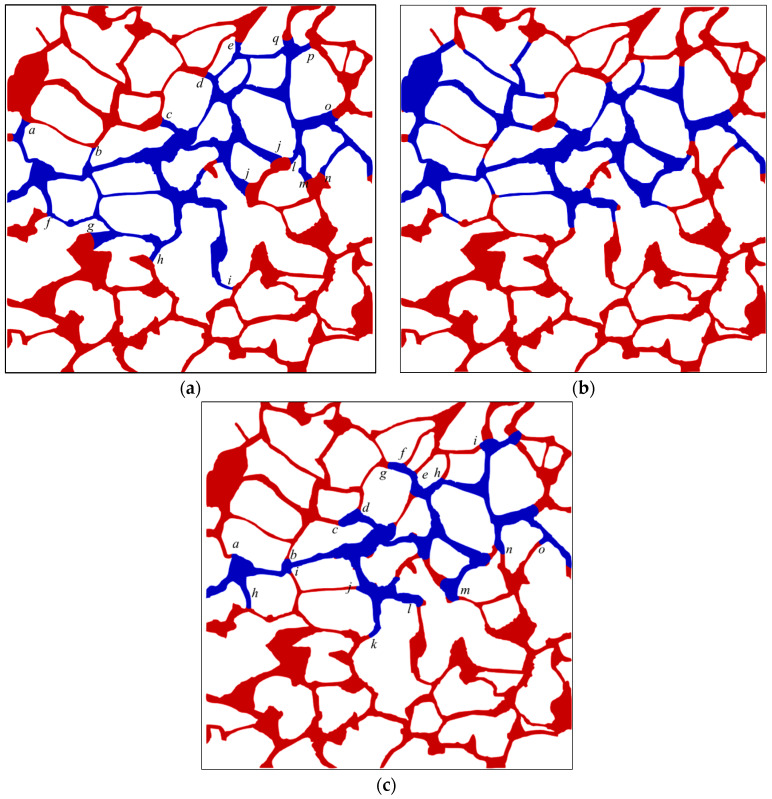
The oil and water distribution under various wettability conditions: (**a**) 30°; (**b**) 90°; (**c**) 150°. (The italic letters (*a*–*q*) denote the interface positions; the oil and water phases are distinguished by red and blue color, respectively.)

**Figure 13:**
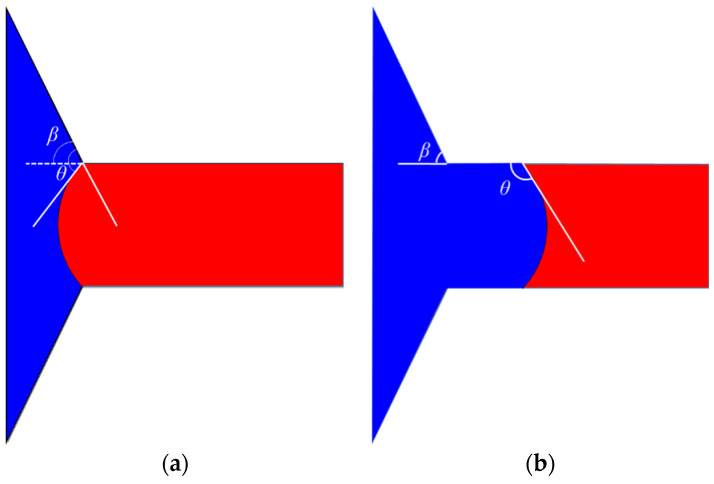
**Scenario I:** the phase distribution before and after the interface entering the throat channel: (**a**) interface is in the pores; (**b**) interface in the throat. (The oil and water phases are distinguished by red and blue color, respectively.)

The final oil–water distribution under different wettability conditions is demonstrated in Appendix C, and the phase distributions under contact angles of 30°, 90°, and 150° are illustrated in Figure 12. In terms of the sweep extent, the spatial sweep area of water under intermediate wettability conditions is the largest, as shown in Figure 12b, while the minimum spatial sweep area of water is observed under strong oil-wet conditions. The interface predominantly remains at the junction from the throat channel to the pore space under water-wet conditions, as indicated by the interface *a*, *b*, *c*, *d*, *e* in Figure 12a. Further, the entire throat channel is occupied with water due to the capillary barrier phenomenon occurring at these interfaces. Once the capillary barrier phenomenon is observed, the interface at that location is in a “dilemma” of whether to advance or recede; that is, the phenomenon will hinder the interface from advancing and the displacement of the wetting phase by the nonwetting phase will occur if the interface starts to recede. In both cases, the resistance characteristics are observed for the interface, while the interface remains at the junction from the pores to the throat channel (i.e., at interface of *a*, *b*, *c*, *d*, and *e*, as depicted in Figure 12c) under oil-wet conditions, resulting in the entire throat channel becoming filled with oil. This phenomenon occurs as a result of the alteration in the capillary force status at the interface under hydrophobic circumstances. Here, we discuss this issue based on the following two scenarios:

**Scenario I:** When *θ* − *β* < 90° (here, *θ* is the contact angle, and *β* denotes the opening angle), the distribution of oil and water before and after the interface entering the throat channel is depicted in Figure 13. In this case, the interface displays a concave shape towards the oil phase side in the pores. The capillary force acts in the direction of displacement, which exhibits driving force characteristics. However, upon entering the throat channel, the interface becomes concave towards the water phase, and the capillary force direction is opposite to that of displacement, presenting resistance characteristics.

**Figure 14:**
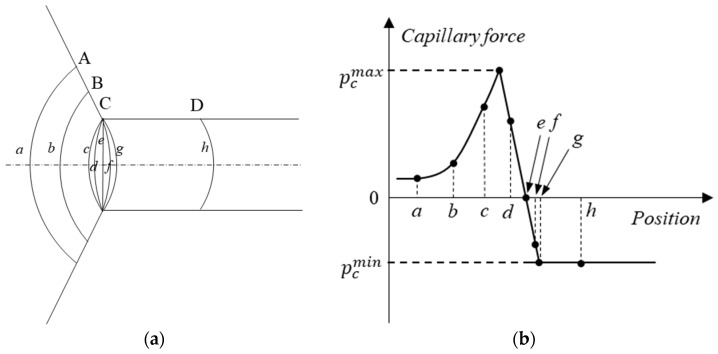
**Scenario I:** the interface shape and the capillary pressure varying with interface positions as the interface advances from the pores to the throat channel: (**a**) the interface at different positions; (**b**) the capillary force at different positions. (The italic letters (*a*–*h*) indicate the oil–water interfaces and the capital letters (A–D) indicate the positions of the interfaces.)

The interface shape and the capillary pressure varying with different interface positions as the interface advances from the pores to the throat channel are shown in Figure 14. As the interface approaches the throat channel, its curvature radius gradually decreases. The capillary force gradually increases as the interface progresses from A to B. When the three-phase contact line advances to position C, the capillary force reaches its maximum value and presents driving force characteristics, which can be expressed as follows

(8) $$p_{c}^{max}=\frac{\sigma cos(\theta -\beta )}{r}$$

where *r* is the throat channel radius.

Upon reaching position C, the three-phase contact line no longer moves, while the oil–water interface shape is continuously changing, and the interface shape continues to evolve from being concave towards the right side of water phase to the left side. As this transition occurs, the interfacial tension gradually changes from driving force to resistance. At the point where the static contact angle emerges between the interface and throat channel, the interfacial tension reaches a maximum negative value, which exhibits resistance characteristics and can be expressed as follows

(9) $$p_{c}^{min}=\frac{\sigma cos(\theta )}{r}$$

It should be noted that, in this scenario, the capillary force undergoes a transition from being a driving force to a resistance as the interface enters the throat from the pores. Further, the same transition of the capillary force can be observed if the interface recedes from the throat to enter the pores. Therefore, the capillary force consistently exhibits resistance characteristics whether the interface is advancing into the throat or receding from it; thus, the interface is in a “dilemma”, which will render the interface immobile at that position in the process of waterflooding.

**Figure 15:**
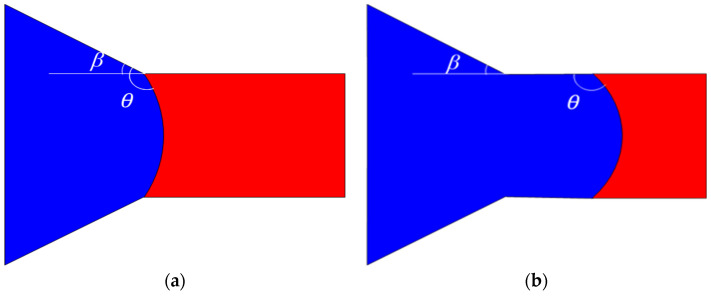
**Scenario II:** the phase distribution before and after the oil–water interface enters the throat channel: (**a**) the interface in the pore; (**b**) the interface enters the throat. (The oil and water phases are distinguished by red and blue color, respectively.)

**Scenario II:** When *θ* − *β >* 90°, the phase distribution before and after the oil–water interface enters the throat channel is shown in Figure 15. As depicted in the figure, the interface displays a concave shape towards the water phase side, irrespective of whether it is situated within the pores or the throat. Consequently, the capillary force presents resistance characteristics during waterflooding.

**Figure 16:**
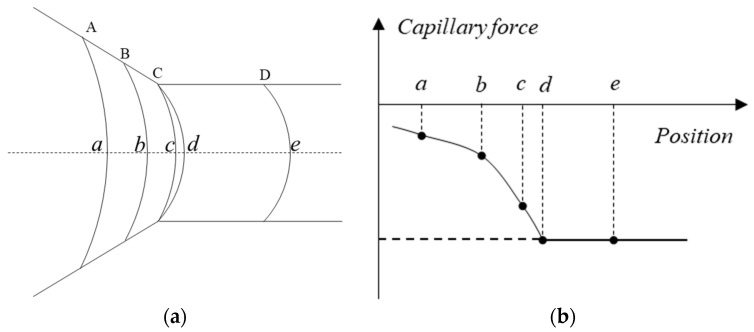
The interface shape and corresponding capillary force varying with interface positions as the interface transitions from the pore to the throat: (**a**) the interface shape varying with positions; (**b**) the capillary force at different positions. (The italic letters (*a*–*e*) indicate the interfaces and the capital letters (A–D) indicate the interface positions.)

The interface shape and the capillary pressure varying with interface positions as the interface transitions from the pore to the throat in scenario II are shown in Figure 16. As the interface advances from position A to position B, entering the throat channel from the pore space, its curvature radius decreases gradually, as illustrated in Figure 16a. Correspondingly, the capillary force gradually increases, as depicted in Figure 16b. The capillary force exhibits resistance characteristics when its value is negative. As the three-phase contact line progresses to position C, the interface shape undergoes deformation while the contact line remains stationary. Upon the formation of the contact angle between the interface and throat wall, the contact line advances from position C to position D, resulting in a minimum value of the capillary force, the magnitude of which can still can be provided by Equation (9).

From the aforementioned analysis, it can be concluded that the capillary force exhibits resistant properties in both **scenario I** and **scenario II**, with maximum magnitude observed once the interface enters the throat section. Therefore, the interface remains at the junction from the pore to the throat.

### 3.4. Analysis of the Seepage Path and Dominant Areas during Waterflooding

#### 3.4.1. Analysis of the Seepage Path during Waterflooding

**Figure 17:**
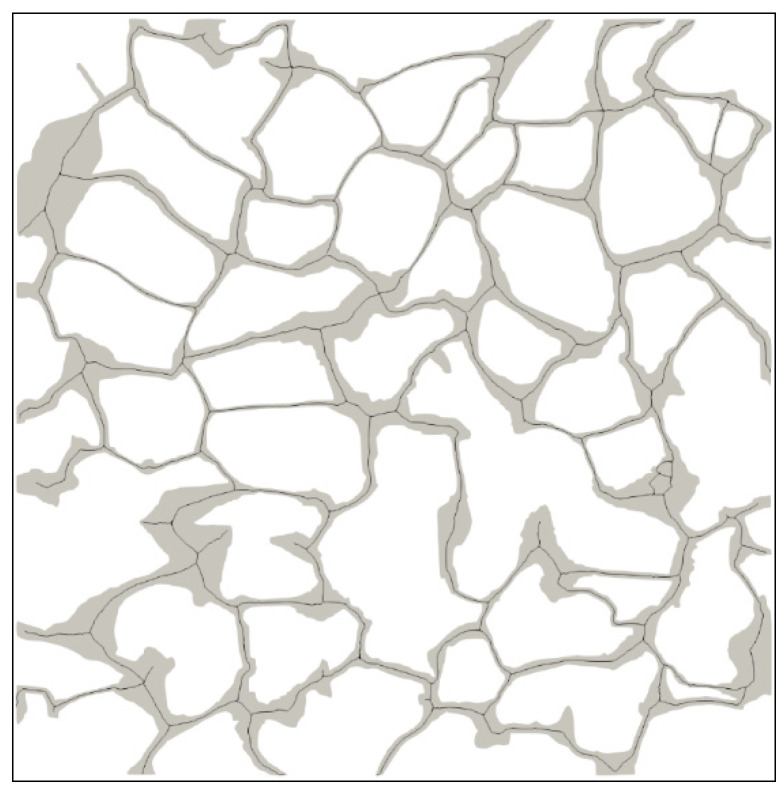
Central axis of the pore network.

**Figure 18:**
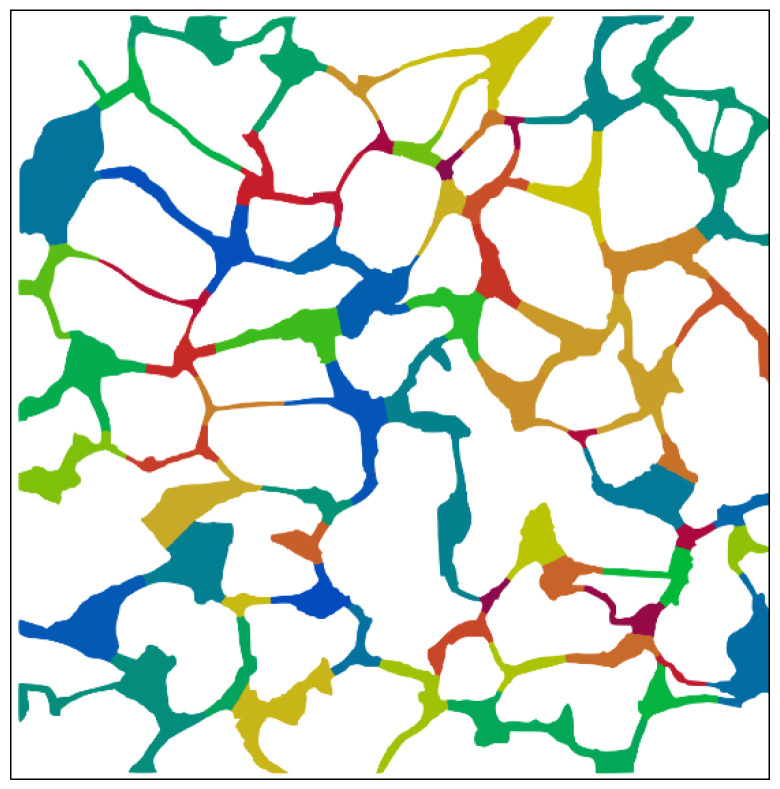
The partition map of the interconnected pore network (different colors indicate different areas).

The central axis of the pore space in digital rock (Figure 5) can be obtained using the approach outlined in Section 2.4.1, as shown in Figure 17. The partition map of the rock can be obtained by segmenting the pore region along its central axis, which is shown in Figure 18.

**Figure 19:**
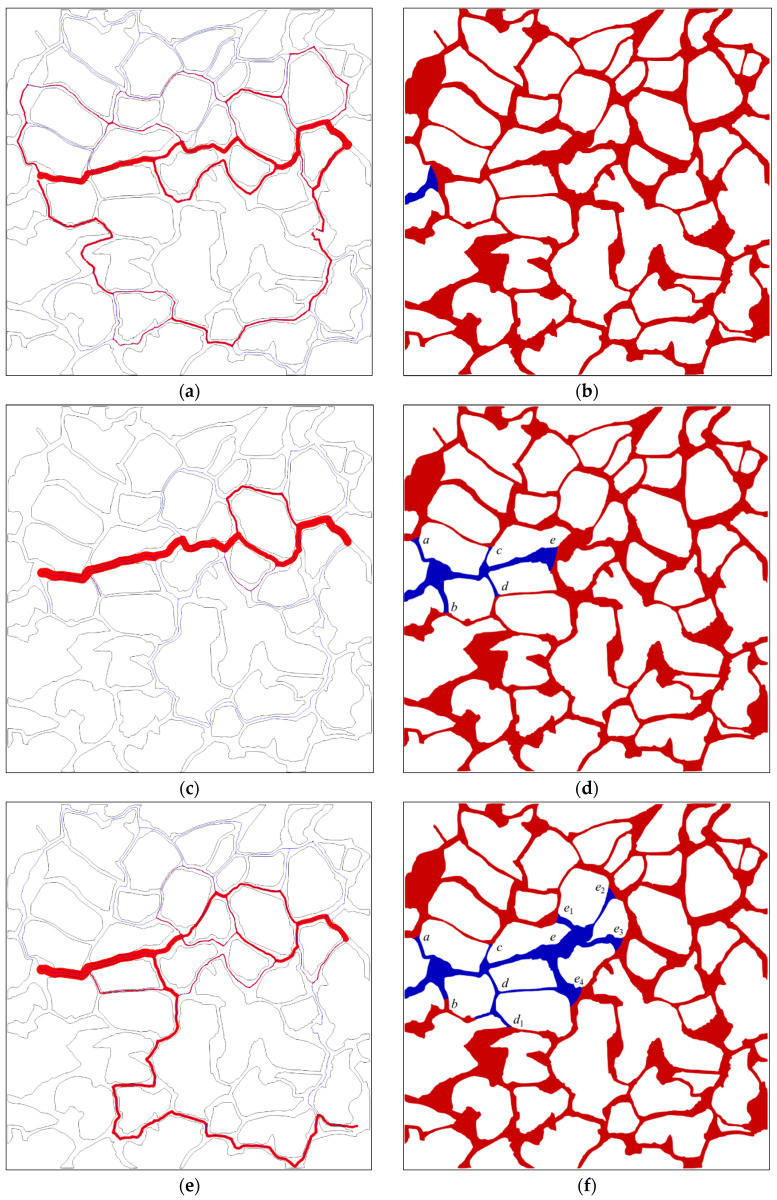

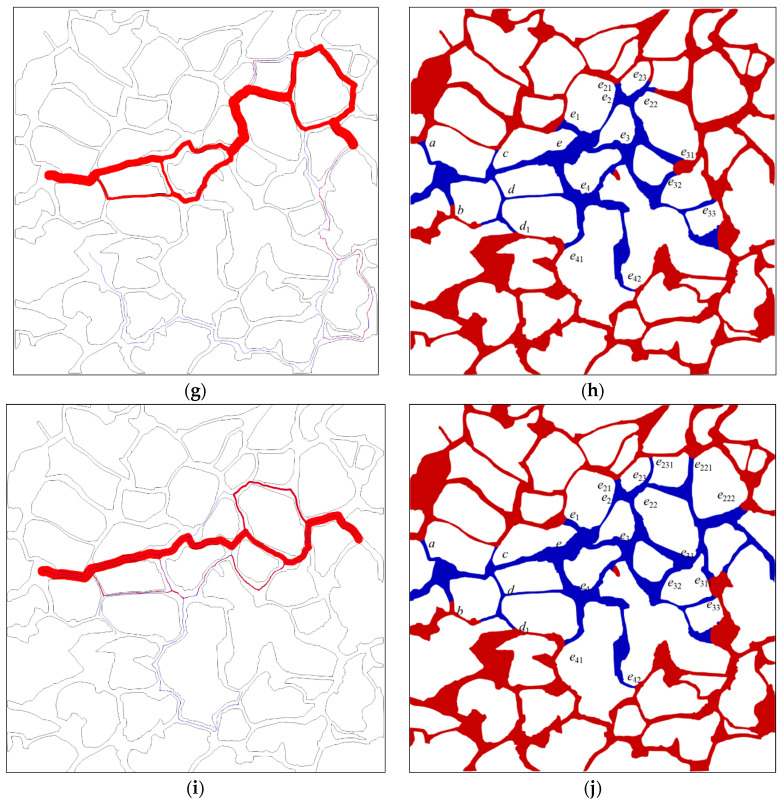
The fluid seepage path at different times and corresponding oil–water distribution at that time: (**a**) seepage path at time 0.01 s; (**b**) oil–water distribution at time 0.01 s; (**c**) seepage path at time 0.05 s; (**d**) oil–water distribution at time 0.05 s; (**e**) seepage path at time 0.1 s; (**f**) oil–water distribution at time 0.1 s; (**g**) seepage path at time 0.2 s; (**h**) oil–water distribution at time 0.2 s; (**i**) seepage path at time 0.3 s; (**j**) oil–water distribution at time 0.3 s. (The blue line in the figure indicates the path with flow rate; the relative magnitude of the flow rate is represented by the thickness of the red line; the thicker the red line, the larger the relative flow rate along that flow path; the italic letters denote the interface positions; the oil and water phases are distinguished by red and blue color, respectively).

The evolution characteristics of the fluid seepage path at the water injection rate of 0.02 m/s are studied here using the particle tracing method coupled with the pore space partition method. The fluid seepage path at different times and corresponding distributions of oil and water over time at each time is displayed in Figure 19. The interface advances into the pore from the injection port at time 0.01 s, as illustrated in Figure 19b. At the moment, other areas of the digital rock are saturated with oil except for the inlet channel. The seepage path shown in Figure 19a reveals that maximum flow rate occurs along the line connecting the inlet and outlet, with a gradual decrease in flow rate along other paths on either side of this line. A fluid flowing phenomenon can be observed at the edge of the rock core, although the flow rate is significantly lower than that within the central channel. Furthermore, the majority of the pore regions in the rock core remain unoccupied by the water phase. The oil–water interface front advanced to positions of *a*, *b*, *c*, and *d* at the intersection of the throat and the pore section at time 0.05 s, as illustrated in Figure 19d; the capillary barrier effect formed at those positions will impede further advancement of the interface. The interface at position *e* will continuously advance and a seepage path will be formed downstream the pore channel from that position. The flow rate within the central channel is significant, whereas, in the other channels, it is relatively low. As the displacement proceeds, the capillary barrier effect occurring at positions of *e*_1_, *e*_2_, and *e*_3_ (as shown in Figure 19f) at time 0.1 s hinders the interfaces at these positions from further advancing. The interface at position e4 advances downward, facilitating the formation of the downward channel displayed in Figure 19e. From phase distribution at time 0.2 s illustrated in Figure 19h, newly formed interfaces at positions of *e*_21_, *e*_31_, *e*_32_, *e*_33_, *e*_41_, and *e*_42_ result in blockage. The interface at position *e*_22_ is in the main channel with large flow rate, which will make the fluid break through along the channel, forming a seepage path, as shown in Figure 19g. With the gradual formation of new oil–water interfaces at positions of *e*_231_, *e*_221_, *e*_222_, and *e*_311_, corresponding channels at these positions are blocked, and a pathway connecting the inlet and the outlet is finally formed. The seepage path finally formed at the end of the waterflooding is shown in Figure 19i.

It can be concluded from the above analysis that the seepage path is directly linked to the capillarity formed during waterflooding. During the initial stage, the displacement process involves the entire fluid within the core, with a limited number of oil–water interfaces present within the pores, while pore channels will be blocked due to the capillary barrier effect when new oil–water interfaces are continuously formed in the pore space, prompting the seepage path to change continuously with positions of the capillary barrier. As the displacement proceeds, the seepage path in the central region is gradually closed by the lateral formed capillary barrier, resulting in a fixed and stable seepage path. The water content of the oil well gradually increases when the oil in that seepage path is gradually recovered.

#### 3.4.2. Analysis of the Dominant Areas during Waterflooding

**Figure 20:**
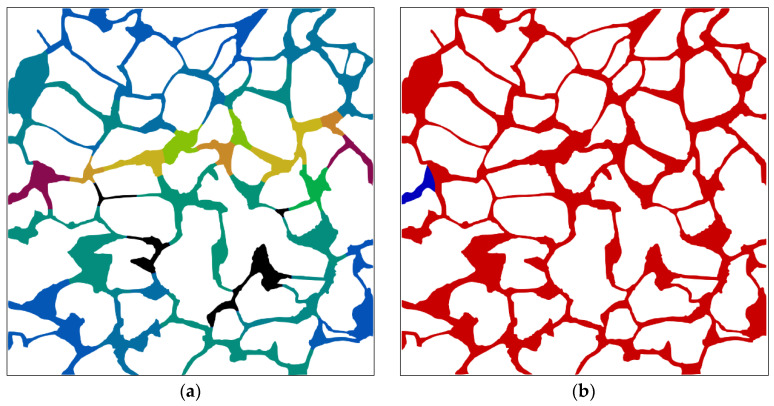

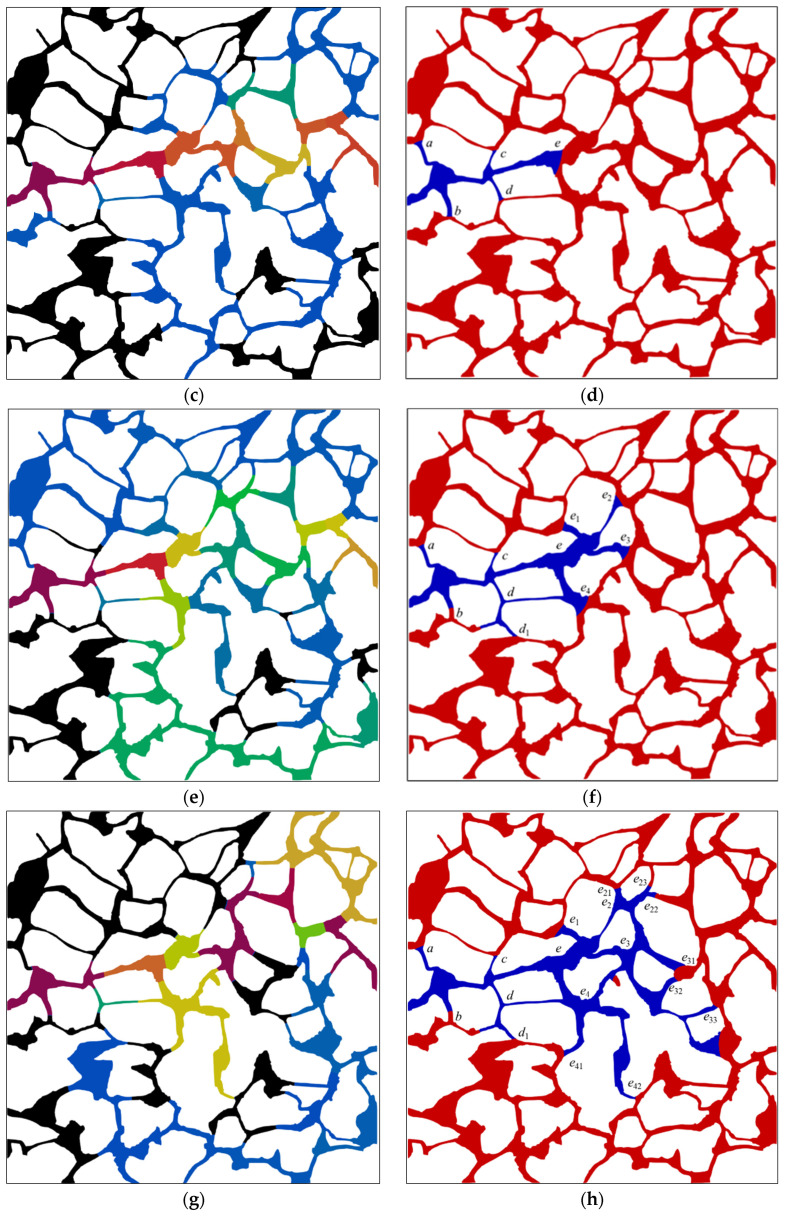

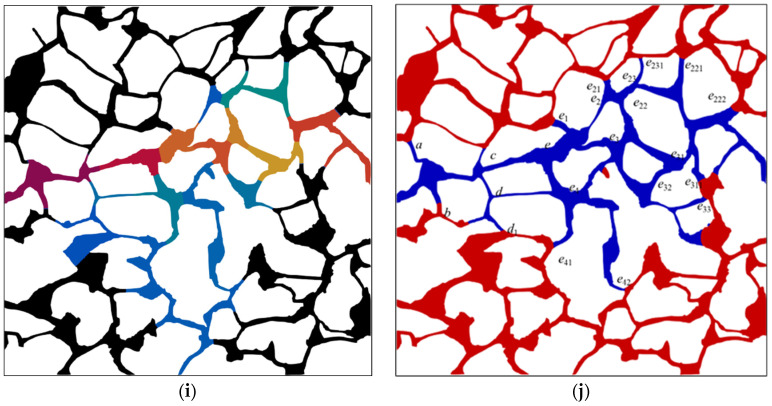
The dominant area and corresponding oil–water distribution at different times: (**a**) dominant area of waterflooding at 0.01 s; (**b**) oil–water distribution at time 0.01 s; (**c**) dominant area of waterflooding at time 0.05 s; (**d**) oil–water distribution at time 0.05 s; (**e**) dominant area of waterflooding at time 0.1 s; (**f**) oil–water distribution at time 0.1 s; (**g**) dominant area of waterflooding at time 0.2 s; (**h**) oil–water distribution at time 0.2 s; (**i**) dominant area of waterflooding at time 0.3 s; (**j**) oil–water distribution at time 0.3 s. (The black area in the figure indicates the fluid is static, and the colored areas indicate dominant areas of the waterflooding; the black area indicates the fluid is static, and the colored area indicates dominant area of the waterflooding; a color gradient ranging from red to blue indicates the relative fluid flow rate, with red implying higher flow rates and blue indicating lower rates; the italic letters denote the interface positions; the oil and water phases are distinguished by red and blue color, respectively).

The dominant areas of the pore network within the digital rock during waterflooding at different times are identified by employing the procedures outlined in Section 2.4.2. As displayed in Figure 20, the dominant area and corresponding oil–water distribution vary with time. It can be observed from the figure that the fluid is almost in motion throughout the entire pore space of the rock at the initial moment (e.g., at time 0.01 s) and the flow is mainly observed in the central region. While there is fluid flowing in the edge area, the velocity is relatively low compared with the velocity in the central region; thus, the oil mobilization efficiency is not high. At time 0.05 s, the capillary barrier effect formed at interfaces at positions of *a*, *b*, *c,* and *d* will hinder the pressure transmission (as shown in Figure 20d), thus preventing the water from flowing through the pore space where interfaces *a*-*d* are located (as illustrated in Figure 20c). The interface at position *e* continues to advance and dominates the pore space downstream the channel where the interface is located (as demonstrated in Figure 20c). However, the fluid flow phenomenon can be obviously observed in the central channel region downstream position *e*, and no significant lateral flow can be observed. The pore space where the interface is located can be used to control the surrounding pores of the interface and mobilize the trapped oil as the interface advances downward, as shown in Figure 20f. Channels at positions of *e*_1_, *e*_2_, and *e*_3_ are in a resistance state, resulting in the lateral flow of the fluid. Therefore, the dominant area of waterflooding expands along the lateral direction. New oil–water interfaces form continuously as the fluid flows downward and the advancement of the interface ceases upon encountering the capillary barrier. The expansion of the dominant area during waterflooding will be impeded by the capillary barrier that emerges at the interface. The capillary barrier occurring at interface *e*_42_ (as displayed in Figure 20h) obstructs the expansion of the dominant area during waterflooding; thus, the fluid of surrounding pore space at that position is not in motion (as shown in Figure 20g). Upon completion of waterflooding, the residual oil in the lateral direction is trapped by the capillary barrier and the dominant area of waterflooding is confined to the water-bearing zone, as shown in Figure 20i.

## 4. Conclusions

In this study, the effect of influencing factors, including the water injection velocities, the viscosity ratios of oil to water, and the wettability conditions, on the flow characteristics of waterflooding within a two-dimensional digital sandstone rock model reconstructed based on the micro-CT technology was systematically investigated employing the direct numerical simulation (DNS) method combined with the volume of fluid method (VOF). The seepage paths and the dominant areas during waterflooding are obtained using the particle tracing method, and the underlying microscopic displacement mechanisms are studied and analyzed to mobilize the trapped oil within the pores. The main findings of this investigation are presented below:

(1) The waterflooding process is primarily governed by the competition between capillary and viscous effect. At low water injection rates, the capillary effect dominates and the oil recovery rate is relatively low, while, at higher rates, the viscous effect takes over, leading to the phenomenon of viscous fingering that results in reduced oil recovery rates. An optimal oil recovery rate can be obtained at an intermediate water injection rate due to the balanced influence of both effects in the process. Further, the capillary barrier phenomenon can only be observed at interfaces at the intersection between the throat and pores.
(2) A negative correlation exists between final oil recovery rate and viscosity ratios, and a low viscosity ratio is unlikely to facilitate the sequestration of residual oil in the channel. After waterflooding, the oil–water interfaces consistently remain located at the intersection of the pore space and throat channel on both sides of the primary water-bearing channel after waterflooding and the characteristic capillary force resulting from the capillary barrier phenomenon determines the movement status of the interfaces. The dynamic inhomogeneity ensuing from the alteration in phase distribution is responsible for the oil recovery rate at different oil–water viscosity ratios, and the magnitude of the inhibition and promotion effects during waterflooding is determined by the ratio of the capillary force to the viscous resistance.
(3) The capillary force can exhibit driving and resistance characteristics under varying wettability conditions during waterflooding. The transition of these two characteristics is determined by the interface position within the pore network. When the capillary barrier phenomenon is observed, the interface at that location is in a “dilemma” of whether to advance or recede, that is, the phenomenon will hinder the interface from advancing, and the capillary force exhibits resistant properties. The maximum magnitude is observed once the interface enters the throat section, and the interface remains at the junction from the pore to the throat, which is a significant reason for the residual oil being trapped. Moreover, the ultimate recovery of oil is 10.6% and 24.7% lower under strong water-wet and oil-wet conditions, respectively, compared to the 32.36% recovery rate under the intermediate wettability conditions. The final oil recovery rate is relatively higher in water-wet conditions than in oil-wet conditions.
(4) The seepage path and the dominant areas during waterflooding are directly linked to the capillary barrier formed at the oil–water interface. The capillary barrier occurring in a lateral direction can close the seepage path formed in the central region, and the capillary barrier can bock the expansion of the dominant areas of waterflooding. By regulating the seepage path and dominant areas dynamically, the residual oil within the pores of the rock core can be mobilized to further enhance the oil recovery rate.

It is true that natural reservoir flow in pore structures is inherently three-dimensional. However, the dynamics of fluid–fluid displacement in porous media are governed by the competition of viscous and capillary forces. As such, the effect of these forces on flow behavior in 3D reservoir flow can also be represented in a 2D model. Interestingly, the conclusions summarized in 2D and 3D pore structures are usually similar. To create a two-dimensional pore structure that accurately reflects the properties of the natural sandstone rock, it is necessary to transform pore morphology information from a 3D domain into a 2D field as much as possible. The obtained 2D pore network model in this paper may not fully capture the characteristics of the 3D natural rock to a large extent. Therefore, we plan to perform 3D simulations of oil–water flow in reservoir rock to take this limitation into account in our future research.

## Figures and Tables

**Table 1:** Numerical boundary conditions.

Boundaries	Field	Boundary Condition Type	Comment
Inlet (A)	Velocity (U)	fixedValue	Alter the value to study the effect of water injection velocity.
	Pressure (p)	zeroGradient	
	Water saturation (alpha)	fixedValue	The value 1 indicates areas with water phase.
Outlet (B)	Velocity (U)	zeroGradient	
	Pressure (p)	fixedValue	
	Water saturation (alpha)	zeroGradient	
Wall	Velocity (U)	fixedValue	The value is set to (0,0,0).
	Pressure (p)	fixedFluxPressure	
	Water saturation (alpha)	alphaContactAngle	Change the value to investigate the influence of the wettability.

**Table 2:** Physical properties of the fluids.

Fluid Media	Physical Properties	Value	Comment
Oil	Kinematic viscosity *υ* (m^2^/s)	6.25 × 10^−6^	Alter the value to investigate the effect of oil–water viscosity ratios.
	Density *ρ* (kg/m^3^)	800	
Water	Kinematic viscosity *υ* (m^2^/s)	10^−6^	
	Density *ρ* (kg/m^3^)	1000	
Oil–water interface	interfacial tension σ (kg/s^2^)	0.07	

**Figure A1:**
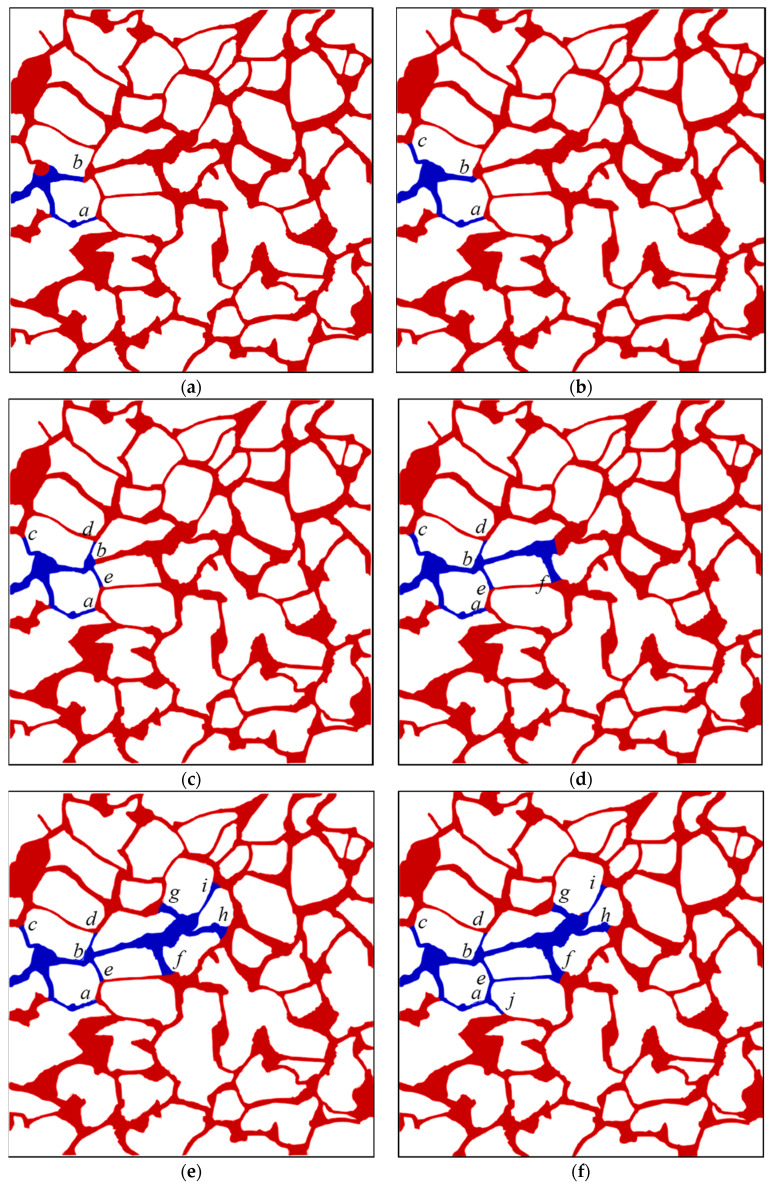

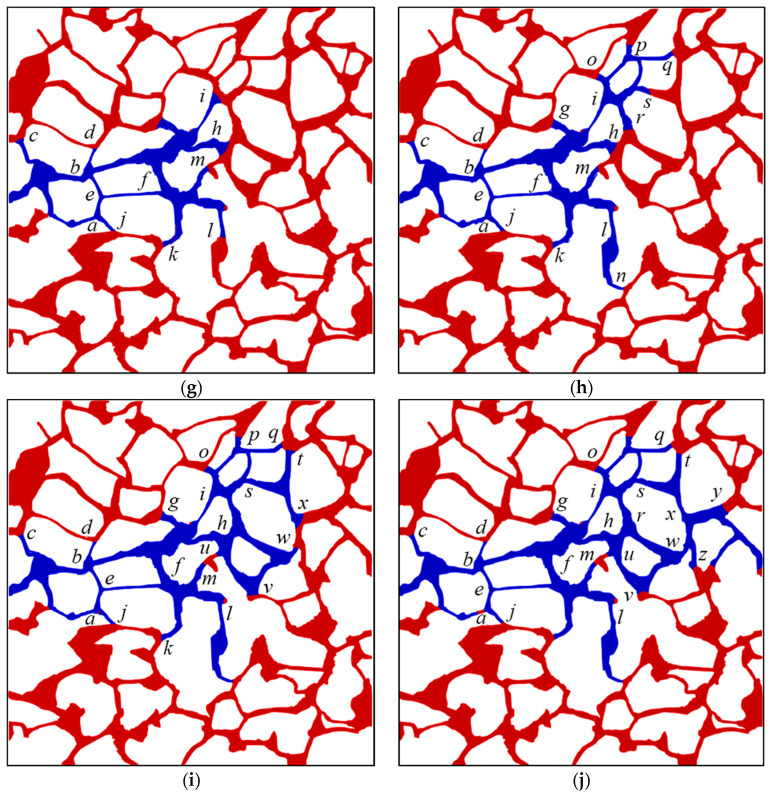
The oil–water distribution and the corresponding interface positions at certain moments: (**a**) 0.5 s; (**b**) 0.65 s; (**c**) 0.75 s; (**d**) 1.2 s; (**e**) 1.8 s; (**f**) 1.9 s; (**g**) 2.125 s; (**h**) 2.25 s; (**i**) 2.45 s; (**j)** final. (The interface positions are designated by lowcase letters in italics; the oil and water phases are distinguished by red and blue color, respectively).

**Figure A2:**
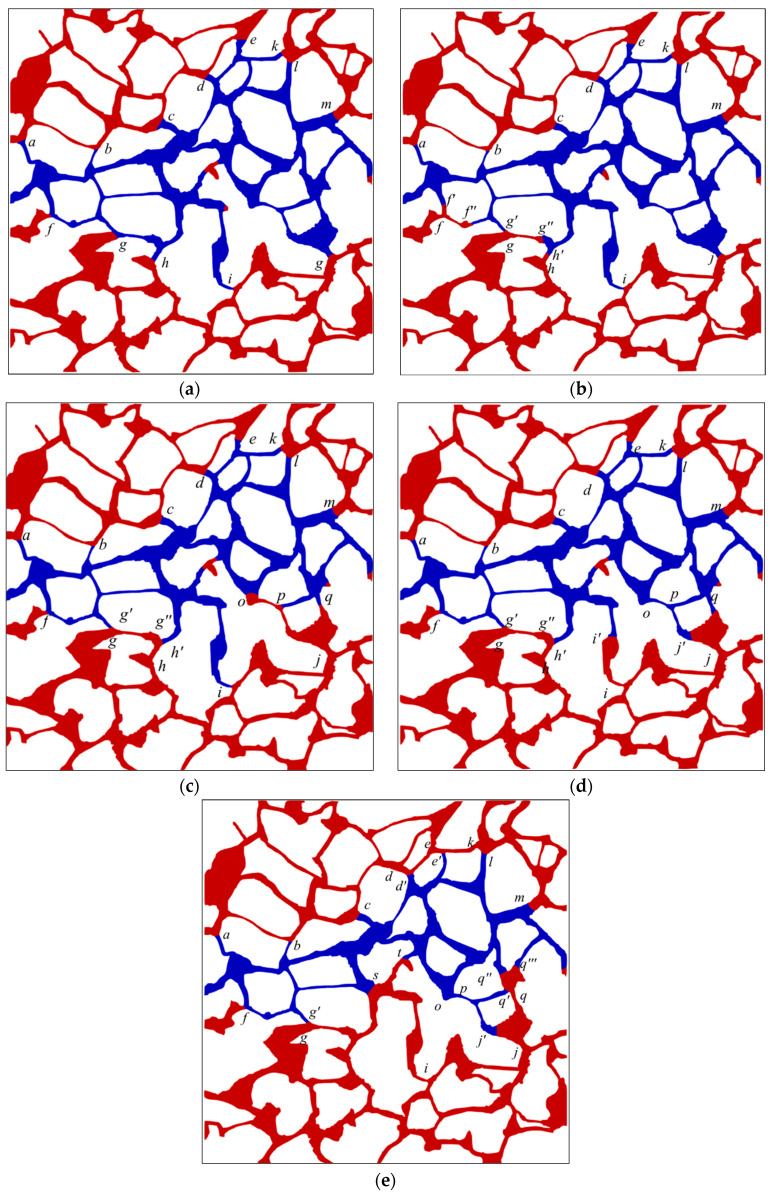
The oil and water distribution after waterflooding (Ca = 7.14 × 10^−5^) under varying viscosity ratios: (**a**) 2; (**b**) 5; (**c**) 10; (**d**) 15; (**e**) 20. (The interface positions are designated by lowcase letters in italics; the oil and water phases are distinguished by red and blue color, respectively).

**Figure A3:**
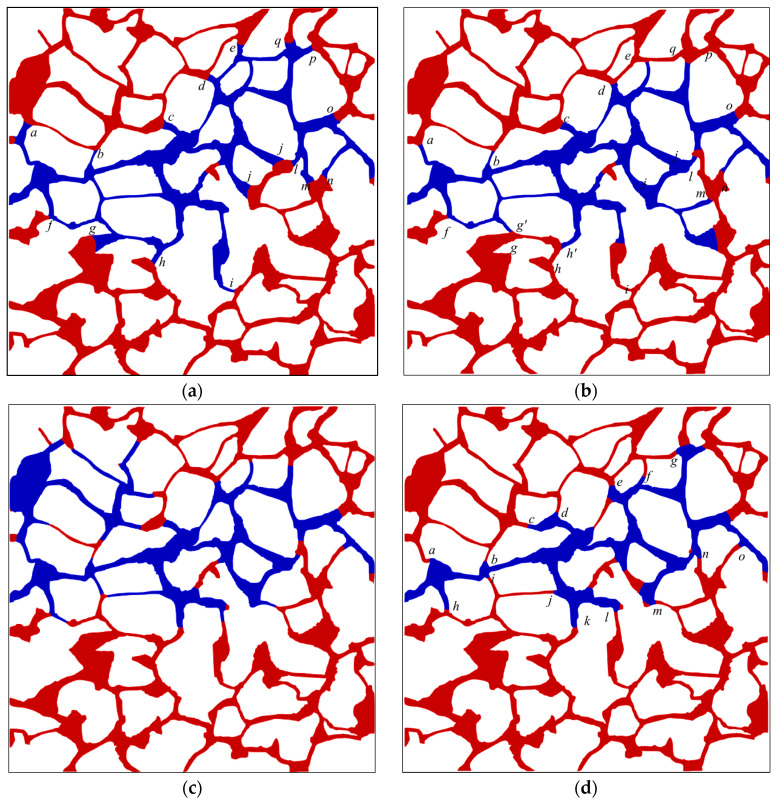

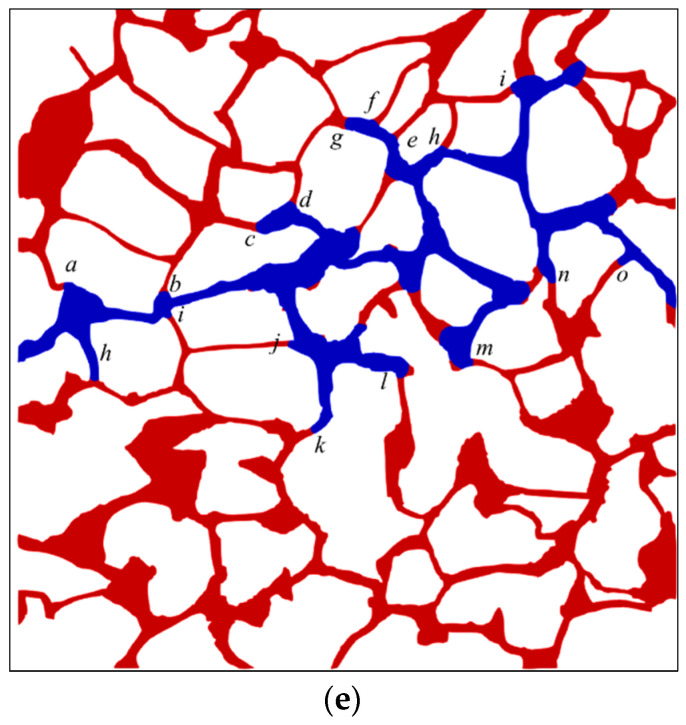
The oil and water distribution under various wettability conditions: (**a**) 30°; (**b**) 60°; (**c**) 90°; (**d**) 120°; (**e**) 150°. (The italic letters (*a*–*q*) denote the interface positions; the oil and water phases are distinguished by red and blue color, respectively).

## Data Availability

Not applicable.
